# Artificial Intelligence in Medical Education: Comparative Analysis of ChatGPT, Bing, and Medical Students in Germany

**DOI:** 10.2196/46482

**Published:** 2023-09-04

**Authors:** Jonas Roos, Adnan Kasapovic, Tom Jansen, Robert Kaczmarczyk

**Affiliations:** 1 Department of Orthopedics and Trauma Surgery University Hospital of Bonn Bonn Germany; 2 Department of Dermatology and Allergy Technical University of Munich Munich Germany

**Keywords:** medical education, state examinations, exams, large language models, artificial intelligence, ChatGPT

## Abstract

**Background:**

Large language models (LLMs) have demonstrated significant potential in diverse domains, including medicine. Nonetheless, there is a scarcity of studies examining their performance in medical examinations, especially those conducted in languages other than English, and in direct comparison with medical students. Analyzing the performance of LLMs in state medical examinations can provide insights into their capabilities and limitations and evaluate their potential role in medical education and examination preparation.

**Objective:**

This study aimed to assess and compare the performance of 3 LLMs, GPT-4, Bing, and GPT-3.5-Turbo, in the German Medical State Examinations of 2022 and to evaluate their performance relative to that of medical students.

**Methods:**

The LLMs were assessed on a total of 630 questions from the spring and fall German Medical State Examinations of 2022. The performance was evaluated with and without media-related questions. Statistical analyses included 1-way ANOVA and independent samples *t* tests for pairwise comparisons. The relative strength of the LLMs in comparison with that of the students was also evaluated.

**Results:**

GPT-4 achieved the highest overall performance, correctly answering 88.1% of questions, closely followed by Bing (86.0%) and GPT-3.5-Turbo (65.7%). The students had an average correct answer rate of 74.6%. Both GPT-4 and Bing significantly outperformed the students in both examinations. When media questions were excluded, Bing achieved the highest performance of 90.7%, closely followed by GPT-4 (90.4%), while GPT-3.5-Turbo lagged (68.2%). There was a significant decline in the performance of GPT-4 and Bing in the fall 2022 examination, which was attributed to a higher proportion of media-related questions and a potential increase in question difficulty.

**Conclusions:**

LLMs, particularly GPT-4 and Bing, demonstrate potential as valuable tools in medical education and for pretesting examination questions. Their high performance, even relative to that of medical students, indicates promising avenues for further development and integration into the educational and clinical landscape.

## Introduction

The minimum duration of study for the medical degree in Europe is 6 years. In Germany, state examinations take place after the second, fifth, and sixth years. The first and second examinations are multiple-choice tests [1]. In the second examination, a total of 320 multiple-choice questions are asked, for which the students have 5 hours on each of the 3 examination days. The questions refer to different clinical scenarios and are asked as either single questions or several consecutive questions. In this examination, theoretical clinical knowledge from prior study is tested. Therefore, questions will be asked about knowledge that can generally be availed of on the internet, textbooks, and scientific publications.

Today, promising applications of large language models (LLMs), particularly ChatGPT, are already being observed in education and research, with the potential to trigger a paradigm shift in health care [2]. New, highly flexible artificial intelligence (AI) models have the potential to contribute to novel capabilities in medicine and ultimately enable advanced medical inferences [3]. 

ChatGPT is a web-based platform using advanced AI-driven LLMs such as GPT-3.5-Turbo and GPT-4 developed by OpenAI [4]. It was trained on a massive corpus of text data, allowing it to generate human-like responses to a wide range of questions and prompts. The model is based on the transformer architecture [5], which has proven to be highly effective in natural language processing tasks. ChatGPT’s underlying models use a deep neural network with multiple layers to generate its responses. These models have been fine-tuned on specific tasks to improve its performance, but they are also capable of learning and adapting to new information over time. In terms of its capabilities, ChatGPT’s models can generate text that is coherent, consistent, and contextually relevant. It can answer questions, generate summaries, write stories, and perform various other language-related tasks. The model's performance has been evaluated using various metrics, and it has consistently demonstrated high levels of accuracy and fluency [6]. However, like other LLMs, ChatGPT suffers from hallucination problems. As it does not have access to an external knowledge base, more extrinsic hallucinations are generated [7].

Compared to GPT-3.5-Turbo, its successor GPT-4 can understand more nuanced instructions and questions and is expected to provide false information less frequently. Moreover, it is more likely to refuse queries that could result in harmful responses [8]. GPT-3.5-Turbo and GPT-4 have been trained on data sets up until approximately September 2021 [9]. The parameter size, a value that describes a model’s size, of GPT-4 is approximately 6 times that of GPT-3.5-Turbo. The training data sets for GPT-3.5-Turbo consist of 93% English-language content [10]. Since it is not connected to the internet, it only has limited knowledge of events or information after this period [11].

Bing is an AI chatbot developed by Microsoft and has been unveiled in 2023. In the development process, technology from GPT-4 was used to enhance its accuracy and performance capabilities [12,13]. In contrast to ChatGPT, Bing AI actively searches the internet for pertinent content and can provide relevant sources for its specific responses [14].

Overall, ChatGPT and Bing represent a significant advancement in the field of AI and natural language processing. The ability to generate human-like responses and adapt to new information make them valuable tools for a wide range of applications, including content creation, customer service, and research.

This study critically examines the capabilities of ChatGPT and Bing in medical education, by addressing how Bing, GPT-4, and GPT-3.5-Turbo perform in answering multiple-choice questions on the German Medical State Examination of 2022. Specifically, we assessed the number of correct answers of these LLMs in German, which is not their main training language, and compared it to the performance of medical students to evaluate their usefulness in the medical field.

## Methods

### Study Design

We conducted a retrospective analysis of the spring and fall 2022 German Medical State Examinations. A total of 630 out of 640 multiple-choice questions in German (including questions containing media) were analyzed. Questions that were excluded post hoc for factual incorrectness were excluded from further analysis (1 from among 320 questions from the spring examination and 9 from 320 questions from the fall examination). The questions were taken from the learning platform Amboss [15]. We used OpenAI’s Python application programming interface [16] to query prompts for the base models of GPT-3.5-Turbo and GPT-4 on June 9, 2023, and the Bing queries were made between the June 9 and 13, 2023, using the custom Python library EdgeGPT (version 0.10.7; Binedge.ai) and the *precise* settings [17]. No data were excluded, unless specifically mentioned. The students’ results were obtained from the Institute for Medical and Pharmaceutical Examination Questions [18].

We asked examination questions based on a German translation of the scheme used in previous studies evaluating OpenAI’s models [19]: “The following are multiple choice questions (with answers) about medical knowledge. {{context}} **Question:** {{question}} {{answer_choices}} **Answer:**(.“

### Statistical Analysis

We used a MacBook M1 pro 14-inch 2021 device with macOS Ventura (version 13.4), with Python (version 3.8.11) installed, and the data analysis libraries numpy (version 1.21.6) and pandas (version 1.4.3). For visualization, we used matplotlib (version 3.5.2) and seaborn (version 0.11.2). One-way ANOVA and independent samples *t* tests were carried out with python statistical library scipy (version 1.7.3) to assess statistical differences between means.

For analysis without media content (eg, questions containing images), 38 out of 311 (12.2%) questions were excluded for the spring examination and 22 out of 319 (6.9%) questions for the fall examination. The models’ strength was calculated as a relative proportion of the number of correct answers provided by students; a value above 100% means that the model was outperforming the average student, whereas a value under 100% shows below average student performance.

When writing this paper, the authors used Grammarly (Grammarly, Inc) and GPT-4 to improve the language of the manuscript and correct grammatical errors. After using these tools, the authors reviewed and edited the content as needed and take full responsibility for the content of the publication.

## Results

We compared the performance of 3 LLMs, GPT-4, GPT-3.5-Turbo, and Bing, in the German Medical State Examinations of 2022, both in spring and fall, and with and without questions containing media. The performance of the models was also compared to that of the students who participated in the examinations.

### Overall Results on Including Questions With Media

GPT-4 had the highest overall performance with 555 correct answers out of 630 (88.1%) questions. Bing followed closely with 542 out of 630 (86.0%) correct answers. GPT-3.5-Turbo lagged with 414 out of 630 (65.7%) correct answers. The students performed in between (469.7/630, 74.6%).

One-way ANOVA revealed significant differences among the 3 models (*F*_2_=64.1, *P*<.001). Independent samples *t* tests were conducted to make pairwise comparisons between the models. There was no significant difference in performance between Bing and GPT-4 (*t*_1258_=–1.09, *P*=.28). However, both Bing (*t*_1258_=8.67, *P*<.001) and GPT-4 (*t*_1258_= 9.77, *P*<.001) performed significantly better than GPT-3.5-Turbo.

Further, statistical analyses were conducted to compare the performances of GPT-3.5-Turbo, GPT-4, and Bing with that of students in the spring 2022 and fall 2022 German Medical State Examinations.

**Figure 1:**
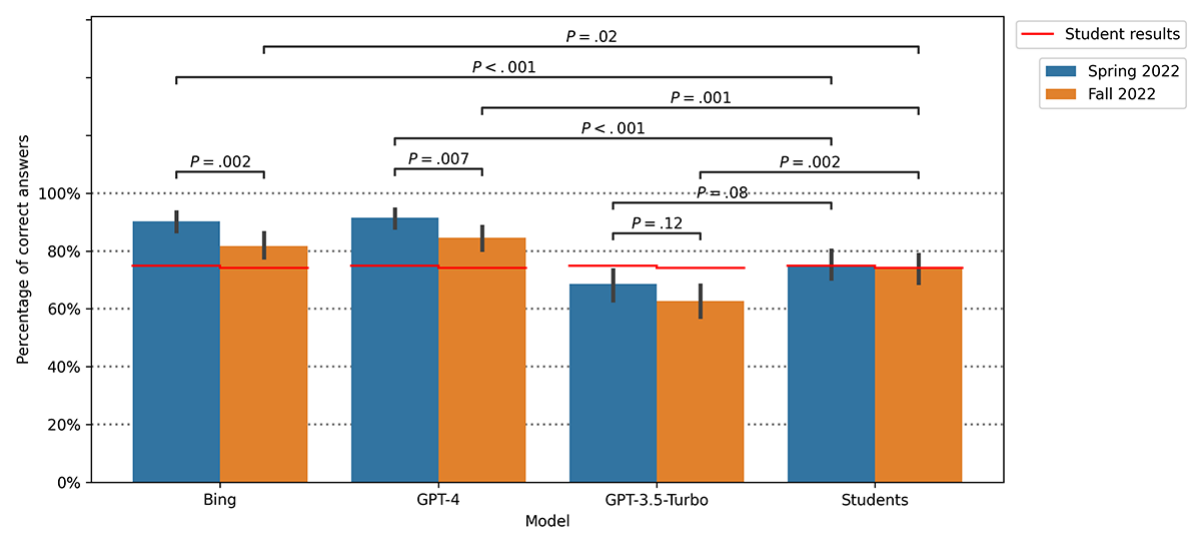
Comparative analysis of the performance of students and 3 large language models (LLMs)—Bing, GPT-4, and GPT-3.5-Turbo—on the German Medical State Examinations conducted in spring and fall 2022. The graph delineates the mean scores for each group, with error bars representing the 95% CIs around the mean. Independent samples *t* tests were used to statistically assess the differences between the students' scores and those of the 3 LLMs. Moreover, a comparison was made between the performances in the spring and fall examinations. The figure effectively displays the relative strengths and variations in the performances of the 3 LLMs and students across the 2 examination periods.

In the spring 2022 examination, GPT-3.5-Turbo’s performance was not significantly different from that of the students (*P*=.08, *t*_636_=–1.76). However, both GPT-4 (*P*<.001, *t*_636_=5.751) and Bing (*P*<.001, *t*_636_=5.22) significantly outperformed the students. In the fall 2022 examination, GPT-3.5-Turbo’s performance was significantly lower than that of the students (*P*=.002, *t*_620_=–3.10). In contrast, both GPT-4 (*P*=.001, *t*_620_=3.23) and Bing (*P*=.02, *t*_620_=2.26) again significantly outperformed the students (Figure 1).

In summary, GPT-4 and Bing demonstrated similar performance levels and significantly outperformed GPT-3.5-Turbo on the set of 630 questions, which included those with image components that are inaccessible to the models. GPT-4 and Bing consistently outperformed students in both the spring and fall 2022 examinations. GPT-3.5-Turbo's performance was comparable to that of students in the spring 2022 examination but was significantly lower in the fall 2022 examination.

### Overall Results Excluding Questions With Media

Next, the 40 questions containing media content were excluded, leaving a total of 590 questions for analysis. Bing achieved the highest performance with 517 correct answers out of 590 (90.7%). GPT-4 was slightly behind, with 515 out of 590 (90.4%) correct answers. GPT-3.5-Turbo, on the other hand, answered 389 out of 590 (68.2%) questions correctly. One-way ANOVA revealed that there were significant differences among the 3 models (*F*_2_=72.8, *P*<.001). Further investigation into these differences was conducted using independent samples *t* tests for pairwise comparisons between the models. There was no significant difference in performance between Bing and GPT-4 (*t*_1178_=0.2, *P*=.84). However, both Bing (*t*_1178_=9.76, *P*<.001) and GPT-4 (*t*_1178_=9.57, *P*<.001) significantly outperformed GPT-3.5-Turbo. Since there are no officially published data on students' performance on individual questions, we were unable to compare the models’ performance on nonmedia questions with that of the students.

In summary, when questions with media content were excluded, GPT-4 and Bing still demonstrated similar performance levels and both significantly outperformed GPT-3.5-Turbo on the set of 590 questions.

### Evaluation Outcomes

A detailed comparison of performance between the models and students, considering questions with and those without media, is shown in Table 1. In the spring 2022 examination, GPT-4 answered 292 out of 319 (91.5%) questions correctly, Bing answered 288 out of 319 (90.3%) questions correctly, and GPT-3.5-Turbo answered 219 out of 319 (68.7%) of questions correctly. When questions with media were excluded, performance improved slightly for Bing (278/297, 93.6%), GPT-4 (276/297, 92.9%), and GPT-3.5-Turbo (208 /297, 70.0%). In contrast, the students achieved, on average, 239 (SD 26.5) correct answers out of 319 (74.9%) questions.

On the fall 2022 examination, both the LLMs and the students performed slightly worse: GPT-4 answered 263 out of 311 (84.6%) questions correctly, Bing answered 254 out of 311 (81.7%) questions correctly, and GPT-3.5-Turbo answered 195 out of 311 (62.7%) questions correctly. When questions containing media were excluded, the performance of all models increased, with Bing having answered 239 out of 273 (87.5%) questions correctly, GPT-4 also having answered 239 out of 273 (87.5%) questions correctly, and GPT-3.5-Turbo having answered 181 out of 273 (66.3%) questions correctly. In comparison, the students answered a mean of 230.7 (SD 22.6) questions correctly out of 311 (74.2%).

**Table 1:** A comparative analysis of the performance of 3 large language models (LLMs), Bing, GPT-4, and GPT-3.5-Turbo, and medical students in the German Medical State Examinations during spring and fall 2022. This table offers insights into how the LLMs performed in contrast to students, considering the inclusion or exclusion of questions with and those without media.

Examination and LLM		Correct answers, n (%)	Total questions, n	Total questions without media, n	Correct answers (without media), n (%)	Correct answers of students, n (%)	Model strength, %	
							With media	Without media
**Spring 2022**								
	Bing	288 (90.3)	319	297	278 (93.6)	239 (74.9)	115.4	118.7
	GPT-4	292 (91.5)	319	297	276 (92.9)	239 (74.9)	116.6	118
	GPT-3.5-Turbo	219 (68.7)	319	297	208 (70)	239 (74.9)	93.8	95.1
**Fall 2022**								
	Bing	254 (81.7)	311	273	239 (87.5)	230.7 (74.2)	107.5	113.3
	GPT-4	263 (84.6)	311	273	239 (87.5)	230.7 (74.2)	110.4	113.3
	GPT-3.5-Turbo	195 (62.7)	311	273	181 (66.3)	230.7 (74.2)	88.5	92.1

### Relative Model Strength Compared to Students

When evaluating the performance of the models relative to that of the students for the spring 2022 examination, GPT-4 displayed a relative strength of 116.6% when including questions with media and 118.0% when including questions without media, whereas Bing demonstrated strengths of 115.4% and 118.7%, respectively. Slightly worse performance, but still exceeding that of the students, was observed for the fall 2022 examination. The relative performance of GPT-3.5-Turbo for both examinations, compared to that of the students, with and without questions containing media, ranged between 88.5% for the fall 2022 examination including questions with media to 95.1% for the spring 2022 examination with questions without media.

### Comparison Between Spring and Fall 2022 Examinations

Statistical analyses of the performances between the spring and fall 2022 examinations revealed significant differences between GPT-4 and Bing. When including questions with media, there were significant differences in performance between the spring and fall 2022 examinations for GPT-4 (+29 correct answers, +6.9%; *P*=.007, *t*_628_=2.71). Furthermore, there were significant differences in Bing’s performance (+34 correct answers, +8.6%; *P*=.002; *t*_628_=3.14). When questions with media were excluded, the differences remained significant, with GPT-4 showing +37 (+5.4%) correct answers (*P*=.03, *t*_568_=2.18) between the spring and fall 2022 examinations. Bing had +39 (+6.1%) correct answers (*P*=.01, *t*_568_=2.5) between the spring and fall 2022 examinations (Figure 1).

In contrast, for GPT-3.5-Turbo, there was no significant difference in performance between the spring and fall 2022 examinations, irrespective of whether questions with media were included (*P*=.12, *t*_628_=1.57) or excluded (*P*=.34, *t*_568_=0.96). The difference in the number of correct answers for GPT-3.5-Turbo was +24 (+6.0%) with and +27 (+5.7%) without questions containing media; however, these differences were not significant.

### Summary of Findings

In summary, both GPT-4 and Bing performed remarkably well on the German Medical State Examinations in 2022, significantly surpassing the performance of students and consistently outperforming GPT-3.5-Turbo. However, GPT-4 had a slight edge over Bing. Furthermore, there were significant seasonal variations in the performance of GPT-4 and Bing but not GPT-3.5-Turbo.

## Discussion

### Principal Results

Overall, all 3 LLMs showed remarkable results in the spring and fall examinations of 2022. GPT-4 and Bing even surpassed the students' scores in both examinations, whereas GPT-3.5-Turbo was just slightly below. In the spring 2022 examination, GPT-4 correctly answered 292 out of 319 (91.5%) questions, and Bing correctly answered 288 out of 319 (90.3%) questions, whereas on average, students correctly answered 239 out of 319 (74.9%) questions. Thus, both models showed outstanding results. A comparison with the highest scoring students revealed that only 0.5% of participants achieved a score between 291 and 300 [18]. Even though GPT-3.5-Turbo lagged behind in score, it still managed to pass the examination. After excluding questions containing media, the performance of all 3 models was further enhanced.

In comparison, both Bing and GPT-4 showed a significant decline in performance in the fall 2022 examination. However, both LLMs were still able to significantly outperform the students and ChatGPT-3.5-Turbo. The performance of GPT-3.5-Turbo and that of the students did not differ significantly between the 2 examinations. Overall, there was a significant difference in performance between Bing and the students in both the spring and fall 2022 examinations, as well as between GPT-4 and the students in both examinations. While GPT-3.5-Turbo was not significantly worse than the students in the spring 2022 examination, a significant difference was observed in the fall 2022 examination. Therefore, not every model appears to be equally suitable for correctly answering medical examination questions. However, a noticeable improvement has been noted with the further developed models. To what extent this can be further improved in the future should be investigated through further comparisons of different models.

### Comparison With Prior Work

An explanation for the poorer performance is the higher proportion of media-related questions in the fall 2022 examination, as there is no image recognition in the current version of the LLMs. This feature is already announced for GPT-4 and needs to be considered in future analyses. The consistent performance of the students and GPT-3.5-Turbo also suggests that there may have been more questions with a higher degree of difficulty in the fall 2022 examination; this also aligns with a current negative trend in the results of the state examinations, with a large variation among individual examinations [20].

Another prominent point in the fall 2022 examination compared to the spring 2022 examination is the relatively high proportion of questions that were subsequently excluded. In the spring 2022 examination, only 1 question was excluded post hoc, whereas 9 were in the fall 2022 examination. Since only those questions that are factually incorrect or whose answer options are contentious were excluded, a high proportion of such questions can lead to uncertainty among students regarding their own decision-making process. Given the outstanding performance of GPT-4 and Bing, it might be worth considering using these LLMs to pretest questions for future examinations to reduce the number of contentious questions. This could be a relevant aspect for further examinations. Bing seems to be a qualified medium, as it can also provide sources for the given answer. The issue remains that incorrect answers from these programs are difficult to detect, especially as the examiner is presented with a seemingly correct solution with explanations. This should be tested in further investigations.

In addition to the actual testing of examinations using the LLMs, the aspect of preparation for these is a crucial point. Besides traditional textbooks, LLMs appear to provide a valuable supplement to conventional learning by elucidating medical issues, offering students the opportunity to obtain rapid solutions for specific medical questions. This also plays a significant role in preparation for examinations. LLMs could be used to quickly inquire about specific medical queries, hence simplifying learning. For instance, extensive research could be made easier by Bing's citation of sources. Future research should investigate how image recognition functions in a medical context to provide, for example, support in radiographic diagnostics or dermatologic findings. ChatGPT is already being increasingly used in the field of radiology, where it can aid in education and assist in making clinical decisions [21]. Especially for young doctors, it could potentially provide an opportunity to facilitate their professional entry through targeted queries. Moreover, there is perceived potential in using LLMs such as ChatGPT as web-based teaching assistants to offer students detailed and relevant information [22].

Certainly, programs using LLMs such as OpenAI’s ChatGPT [4], Microsoft’s Bing [14], and Alphabet’s Bard [23] and PaLM 2 [24] will be further developed and improved in the future and thus be able to provide professionals with well-founded professional answers and lower error and hallucination rates.

### Limitations

In this study, the evaluation was limited to OpenAI's models and focused on single prompts and answers from just 1 year’s German Medical State Examinations. The inability of the models to process media content and the lack of diversity in examination content and languages confines the scope of insights. Additionally, the rapid evolution of LLMs means that the results may quickly become outdated. Moreover, there are implications regarding the accuracy of the LLMs’ outputs and the level of trust that should be placed in them, particularly in the context of medical education. The study did not investigate the potential for misinformation or inaccuracy in the responses generated by the LLMs, which is critical given that medical students might rely on these tools for preparation for examinations. Lastly, potential biases or errors intrinsic to the models were not explored. These constraints warrant measured interpretation of the results and indicate the need for more extensive and varied studies, as well as a critical analysis of the reliability of the LLMs in a medical education setting. 

### Conclusions

Being the fastest growing web platform ever [25], LLMs will attract even more users following Microsoft’s GPT-4 integration into the Edge browser [26]. To better assess the performance of LLMs such as GPT-4 and Bing in medical state examinations, further studies on their performance on older examination questions with more languages are crucial. Equally, studies should investigate how LLMs can respond to specific medical queries independent of given answer options, to further establish them in clinical practice.

## Abbreviations

AI: artificial intelligence
LLM: large language model
